# Measuring the performance of interprofessional primary health care teams: understanding the teams perspective

**DOI:** 10.1017/S1463423619000409

**Published:** 2019-08-28

**Authors:** Catherine Donnelly, Rachelle Ashcroft, Amanda Mofina, Nicole Bobbette, Carol Mulder

**Affiliations:** 1 School of Rehabilitation Therapy, Occupational Therapy Program, Queen’s University, Kingston, ON Canada; 2 Faculty of Social Work, University of Toronto, Toronto, ON Canada; 3 Quality Improvement Decision Support Program, Provincial Lead, Association of Family Health Teams of Ontario, Toronto, ON Canada

**Keywords:** indicators, interprofessional collaboration, interprofessional primary health care teams, performance measurement, primary health care

## Abstract

**Aim:**

The aim of the study was to describe practices that support collaboration in interprofessional primary health care teams, and identify performance indicators perceived to measure the impact of this collaboration from the perspective of interprofessional health providers.

**Background:**

Despite the surge of interprofessional primary health care models implemented across Canada, there is little evidence as to whether or not the intended outcomes of primary health care teams have been achieved. Part of the challenge is determining the most appropriate measures that can demonstrate the value of collaborative care. To date, little remains known about performance measurement from the providers contributing to the collaborative care process in interprofessional primary care teams. Having providers from a range of disciplinary backgrounds assist in the development of performance measures can help identify measures most relevant to demonstrate the value of collaborative care on the intended outcomes of interprofessional primary care models.

**Methods:**

A qualitative study; part of a larger mixed methods developmental evaluation to examine performance measurement in interprofessional primary health care teams. A stakeholder workshop was conducted at an annual association meeting of interprofessional primary health care teams in the province of Ontario, Canada. Six questions guided the workshop groups and participant responses were documented on worksheets and flip charts. All responses were collected and entered verbatim into a word document. Qualitative analytic strategies were applied to each question.

**Findings:**

A total of 283 primary health care providers from 14 health professions working in interprofessional primary health care teams participated. Top three elements of interprofessional collaboration (total *n* = 628) were communication (*n* = 146), co-treatment (*n* = 112) and patient-based conferences (*n* = 81). Top three performance indicators currently used to demonstrate the value of interprofessional collaboration (total *n* = 241) were patient experience (*n* = 71), patient health status (*n* = 35) and within team referrals (*n* = 30).

## Background

Primary health care is the pillar of the health care system, with interprofessional collaborative teams being increasingly considered a key tenet and an important means for providing quality primary health care services (Starfield *et al.*, 2005; Samuelson *et al.*, 2012; Pullon *et al.*, 2016. While interprofessional collaboration is recognized as an essential aspect of care for patients with complex and chronic conditions (Reeves *et al.*, 2011; Pullon et al., 2016), the evidence remains in its infancy. For these reasons, policy makers in Canada and elsewhere have continued to call for greater integration of interprofessional team-based primary health care and the development of indicators that specifically recognize the value of this added lens (Hutchison *et al.*, 2011; Ministry of Health and Long-Term Care, 2015).

Although Reeves *et al*. (2011) demonstrate the variations that exist in conceptualizing interprofessional collaboration in primary health care, it can be understood as the integration and cooperation of different health care professionals that complement one another’s competencies, practices, and skills, thus resulting in optimal use of health care resources (Supper *et al.*, 2014. In primary health care, this means that family physicians and/or nurse practitioners come together with other health care providers such as social workers, occupational therapists, dietitians, pharmacists, and others, in order to provide a broad range of services and programs (Ambrose-Miller and Ashcroft, 2016). Approximately 40% of Canadians have access to interprofessional collaborative teams and individuals with multiple chronic conditions are most likely to receive team-based primary health care (Zygmunt and Berge, 2014). Internationally there is also a strong move to team-based primary health care, often referred to as medical homes, to enhance integration of services and emphasize health promotion and chronic disease management (Hutchison *et al.*, 2011; Naccarella *et al.*, 2013). The evidence to date suggests that interprofessional collaboration contributes to the structures and processes of primary health care, including patient satisfaction and access to care (Donabedian, 2005; Hogg *et al.*, 2008), however, limited knowledge exists about the impact that interprofessional collaborative teams have on patient health outcomes (Paradis and Whitehead, 2018).

Despite the surge of team-based models across Canada, there is relatively little evidence as to whether or not the intended outcomes of interprofessional collaborative teams have been achieved (Spenceley *et al.*, 2013; Ashcroft, 2014; Glazier *et al.*, 2015; Zygmunt and Berge, 2014). Performance measurement is an important part of understanding and improving primary health care, yet the challenge remains as to how best to measure the value of interprofessional collaborative teams. Little knowledge exists about indicators that can help demonstrate the impact on quality of care outcomes for patients receiving interprofessional collaborative primary health care (Thannhauser *et al.*, 2010). Furthermore, there is some evidence that performance measures being used in primary health care settings may even be inappropriate for interprofessional teams, resulting in unintended consequences (Ashcroft, 2014). One of the reasons may be the complexity of practice contexts, variations of the types of providers that might be involved in collaborative care practices, and the broad range of programs and care associated with primary health care (Levitt *et al.*, 2014). Instead, indicators largely focus on organizational structures, collaborative processes, and patient experiences (Levitt *et al.*, 2014; Samuelson *et al.*, 2012). Ontario, Canada, is one province that has substantially invested in integrating interprofessional collaboration in primary health care; however, performance indicators used to determine impact of these team practices are largely based on physician-focused data (Jaakkimainen *et al.*, 2011) with limited to no inclusion of data representative of other interprofessional health providers involved in patient care. Not only is there a gap in knowledge about the impact of these newer interprofessional teams, applying indicators developed for a solo-provider primary care context may be harmful to successful interprofessional collaboration (Ashcroft, 2014).

The purpose of our study was to (1) describe current practices that support interprofessional primary health care and (2) identify current performance indicators measuring the impact of collaboration in interprofessional primary health care teams from an interprofessional health provider perspective.

## Methods

This study was part of a larger developmental evaluation being completed with interprofessional primary health care teams in the province of Ontario, Canada. Developmental evaluations are an approach that is ideal for contexts that are dynamic and can support the ongoing evolution of programs (Patton, 2010). The evaluation has included multiple strategies to engage stakeholders for the purposes of development and improvement. For this study, we are reporting on a stakeholder consultation meeting, which is an exploratory descriptive component of the larger developmental evaluation (Miles *et al.*, 2014). ‘Stakeholders’ are defined as those with a vested interest in an activity and its outcome (Leviton and Melichar, 2016). The stakeholder consultation meetings that we are reporting on in this study took place immediately before the commencement of the 2016 annual meeting of a provincial association of primary health care providers in Ontario.

Facilitated stakeholder meetings are an effective method to engage health care providers on a range of topics related to the structures and processes of health care (Leviton and Melichar, 2016; Mercer *et al.*, 2009; Protheroe *et al.*, 2009; Hand *et al.*, 2011). Participants invited to attend the stakeholder consultation meetings were interprofessional health providers employed in primary health care teams in Ontario, Canada, and who were members of the professional association holding the annual meeting. Fourteen professional groups were represented including, but not limited to, nurses, social workers, pharmacists, physical therapists, occupational therapists, quality improvement specialists, and administrators. Ethics approval was received from the local University Office of Research Ethics (Protocol #31773).

### Data collection

Data collection was completed in small groups composed of participants from a similar professional background. Participants were seated with their designated professional group and were provided with five structured questions that aimed to elicit information on outcomes and indicators used to measure the value of interprofessional care. Nominated leads representing each of the 14 interprofessional health providers’ professional groups helped collaboratively develop the five questions in advance of the stakeholder meeting. The development of the questions was guided by the literature on interprofessional collaboration and team-based primary health care (Gocan *et al.*, 2014), yet were also developed in a way that was relevant for the stakeholders and their context. Through teleconference meetings, the nominated leads and the stakeholder meeting facilitators achieved consensus on the questions that were used to explore stakeholders’ perspective on the current practices that support interprofessional primary health care, and performance indicators that can measure the impact of collaboration in interprofessional primary health care teams.

The stakeholder meetings were led by a team of eight experienced facilitators and researchers in the areas of interprofessional care that included the five co-authors and three additional research assistants. Due to the number of participants, six stakeholder meetings occurred simultaneously in separate rooms. Participants completed the workshops independently and came together for a final summary discussion.

Each room was set up so that participants were sitting at round tables with approximately 8–10 individuals per table. Each stakeholder meeting facilitator had a designated script that they read out-loud to begin the session, the set of five questions to guide small group discussion, clearly defined objectives to ensure consistency in the facilitation across the different professional groups, and worksheets to guide the discussion and documentation. Each stakeholder meeting was three hours in duration and ran from 09:00am to 12:00pm. The first half of the workshop sought to identify outcomes, assessment, and/or indicators currently being used in their interprofessional teams. Questions were introduced sequentially by the facilitator, followed by time for the participants to discuss each question within the smaller tables (see Appendix A). Participants documented their discussion on worksheets and larger flip sheet paper provided to each table by the facilitators. Following each question, there was a brief opportunity for groups to present the results of their discussion back to the larger group. The facilitators kept notes of the large group discussion. All artifacts were maintained for analysis.

The terms outcomes, assessment, and indicators were used in order to be as inclusive as possible to all ways in which teams describe approaches to measuring and demonstrating the value of team-based primary health care. All professions participated in this component of the workshop. The second half of the workshop focused on future possibilities of outcomes and indicators that participants might recommend as an option to demonstrate the value of interprofessional teams on patient outcomes. Two professional groups (nursing and pharmacy) did not participate in the second half due to a pre-scheduled education session, thus, opting out of participating in the final discussion about recommendations.

### Data analysis

Given that a developmental evaluation by design allows for methodological flexibility (Patton, 2010), the primary investigators employed both an initial conventional content analysis approach to identify key themes from the data, as well as engaged in a subsequent rank order of the frequency of codes to help analyze the results (Hsieh and Shannon, 2005). The combination of both identifying key themes, followed by a frequency count, intends to help elucidate both the descriptive thematic categories identified in data, and strives to demonstrate the importance of these categories across diverse professional perspectives. This approach has been effectively employed by other researchers to analyze data from stakeholder meetings (Hand *et al.*, 2011; Mercer *et al.*, 2009; Protheroe *et al.*, 2009).

Beginning with the first question, two of the authors (A.M., N.B.) read the responses from all the professions and developed a set of preliminary codes. The authors (A.M., N.B.) then completed a second reading and independently applied the preliminary codes to the data. They (A.M., N.B.) reviewed the applied codes for agreement, and a third author (C.D.) was consulted when consensus was unable to be attained. As data analysis progressed additional codes emerged, and the initial codes were revised, and some codes were collapsed. For example, a code-entitled communication was created to include both formal (meetings) and informal communication (hallway consults). This process was systematically applied to each of the five questions for all professions. A final review of all the generated codes was completed to ensure clarity and consistency. The codes were then organized into tables to capture the frequency of codes across professions for all five questions.

## Results

A total of 283 participants from 14 professions participated including nursing, administrators, social work, occupational therapy, physical therapy, data management specialists, dietitians, pharmacists, chiropodists, health promoters, nurse practitioners, physician assistants, respiratory therapists, and chiropractors. Twelve unique characteristics were identified that were seen to support interprofessional collaboration in the primary care teams. The top three characteristics (total number of response = 628) were communication (*n* = 146), co-treatment (*n* = 112), and patient-based conferences (*n* = 81) (for details refer Table 1). The top three outcomes, assessments, and/or indicators that were identified as being used to demonstrate the *value of interprofessional collaboration* (total *n* = 241) were patient experience (*n* = 71), patient health status (*n* = 35), and intra-agency team referrals (*n* = 30). Quality improvement was identified as an outcome, assessment, and/or indicator, by a total of 57 participants, but was removed from final analysis as it was felt that quality improvement represents a process in which teams can demonstrate value, rather than a measure itself (for complete details, refer Table 2). The top three performance indicators demonstrating the *value of individual providers’ professional contribution within the team* (as opposed to collaborative interprofessional contribution) (total *n* = 189) were workload measurement (*n* = 43), patient experience (*n* = 40) and patient health status (*n* = 34) (for details, refer Table 3). Quality improvement was identified by a total of 76 participants but was again removed from final tally in this category as it was felt to represent a process versus an outcome indicator.

**Table 1.** Characteristics that support collaboration in primary care teams

Dimensions	Number of responses (*N* = 628) *n* (%)
Communication	146 (23.2)
Co-treatment	112 (17.8)
Patient-based conferences	81 (12.9)
Attitudes/values/beliefs	75 (11.9)
Intra-agency referrals	46 (7.3)
Electronic medical record technology	41 (6.5)
Interprofessional education	29 (4.6)
Inter-sectoral linkages and partnerships	29 (4.6)
Leadership	25 (4.0)
Quality improvement initiatives	21 (3.3)
Location and physical space	16 (2.5)
Human resource support	7 (1.1)

**Table 2.** Measures, outcomes and indicators reported currently being used to demonstrate the value of *interprofessional collaboration* in primary care teams

Measures/outcomes/indicators	Number of responses (*N* = 241) *n* (%)
Patient experience	71 (29.5)
Patient health status	35 (14.5)
Intra-agency referrals	30 (12.4)
Workload measurement	22 (9.1)
Staff experience	21 (8.7)
Co-treatment	17 (7.1)
Access to team-based care	13 (5.4)
Communication	12 (5)
Disease tracking	6 (2.5)
Team processes	6 (2.5)
Patient care plan	5 (2.1)
Intersectoral linkages/partnerships	3 (1.2)

**Table 3.** Measures, outcomes, and indicators reported currently being used to demonstrate the value of *Individual Professional* contribution within the team

Measures/outcomes/indicators	Number of responses (*N* = 189) *n* (%)
Workload measurement	43 (22.6)
Patient experience	40 (21.1)
Patient health status	34 (17.9)
Access to team-based care	24 (12.6)
Staff experiences	17 (8.9)
Referrals	10 (5.3)
Care coordination	9 (4.8)
Peer feedback	7 (3.7)
Patient goals	4 (2.1)
Co-treatment	1 (0.5)

**Figure 1.**
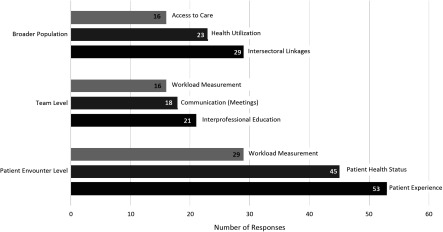
Profession specific future measures of interprofessional collaboration at the population, team and patient encounter level

**Figure 2.**
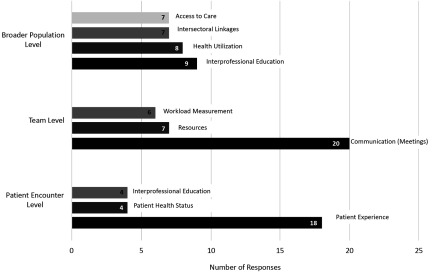
General measures of future interprofessional collaboration at the population, team, and patient encounter level

When participants were asked to identify *potential* performance-specific measures at the level of the individual patient, the team and broader population in order to demonstrate the value of their professions contribution they identified a total of 14 indicators. The top three responses are presented in Figure 1. Teams were also asked to identify *potential* performance-specific measures at the level of the individual patient, the team and the population level to demonstrate the value of collaboration within interprofessional primary health care teams. A total of 14 unique outcomes, assessments, and/or indictors were identified, and the top three responses are presented in Figure 2.

## Discussion

This study provides a unique contribution to the literature on performance measurement of interprofessional primary health care teams by providing the provider perspective on both the existing indicators being used, as well as recommendations for alternative indicators that might better capture collaborative contributions and impact on patient outcomes. The development of performance measurement in primary health care is traditionally completed using top-down approaches such as expert panels (Canadian Institute of Health Information, 2016), literature reviews (Langton et al., 2016), and arm’s-length organizations (Health Quality Ontario, 2016). Top–down approaches to performance measurement are particularly challenging for interprofessional primary health care team members who feel distanced from indicators that are largely biomedical in nature and do not cover the comprehensive attributes and services by non-medical team members (Wong et al., 2011; Ashcroft, 2014; Johnston *et al.*, 2011). There is also evidence to demonstrate that when primary health care teams identify their own performance measures and are able to receive direct feedback on these measures, they are likely to integrate this feedback to adjust practice behaviors (Donnelly *et al.*, 2016). Examples such as this reinforce the need to engage interprofessional health providers in determining meaningful and actionable indicators to demonstrate the value of team-based primary health care. Work is being conducted by the Association of Family Health Teams of Ontario, an organization that represents interprofessional primary health care teams in the province of Ontario, Canada, to engage interprofessional primary health care providers in the identification of performance measures for primary health care teams (Association of Family Health Teams of Ontario, 2017).

### Characteristics of collaboration

In our study, participants identified the characteristics of collaboration they experienced within their interprofessional primary health care teams. These elements were consistent with what has been previously reported in both team-based primary health care (Donnelly et al., 2013; Gocan *et al.*, 2014; Morgan *et al.*, 2015; Saint-Pierre *et al.*, 2018) and the broader literature on collaborative practice (Reeves *et al.*, 2017). The findings of this study add to this growing body of literature on collaborative practice that has clearly identified the importance of team processes (e.g. team meetings), organizational supports (e.g. electronic medical records), and environmental structures (e.g. co-location) in supporting interprofessional primary health care teams.

### Performance indicators

Although both the literature and the results identify processes known to support teams, the actual extent to which these processes are enacted is less clear. There is also relatively little research that has examined the impact of the elements of collaboration on patient outcomes (Brandt *et al.*, 2014; Morgan *et al.*, 2015; Reeves *et al.*, 2017), despite a growing focus on the importance of a team-based approach to managing complex and chronic conditions. Importantly, of the top 10 assessments, outcomes, and/or indicators identified in this study, seven would be considered process indicators (e.g. communication, co-visits, referrals) supporting collaboration. Both this professional readiness and the literature strongly point to the need to include a characteristic(s) of collaboration (e.g. number of team meetings, percentage of the team with access to a common electronic medical record, number of team members who are co-located) as a performance indicator(s) for team-based primary health care. This addition would not only provide a raw team function score, it would also offer insights into the relationship between collaborative processes and patient outcomes.

In Canada, the Canadian Institute of Health Information (CIHI) has developed the Pan-Canadian Primary Health Care Indicators, with a total of 51 indicators, of which 27 indicators are for primary health care policy makers and 24 indicators have been identified for use by practitioners (Canadian Institute for Health Information, 2016). Table 4 provides an overview of the 24 indicators juxtaposed with the outcome/measures/indicators identified in this study. Of note is the heavy focus on coordination outcomes found in this study. One of the CIHI domains, coordination, does in fact address team collaboration; however, there have been no published results related to this indicator, despite data for other key indicators. This lack of data highlights the challenges of collecting data on team processes. Given that ‘coordination’ is one of the few indicators to which non-physician team members directly contribute, both researchers and policy makers need to attend to how a process indicator such as team coordination might be measured consistently.

**Table 4.** Canadian Institute of Health Information (CIHI): Pan Canadian Primary Health Care priority indicators for providers compared to study results

CIHI domain	CIHI indicator	Measures, outcomes and indicators to demonstrate value of interprofessional collaboration	Measures, outcomes and indicators to demonstrate the value of individual professional contribution
Acceptability	Primary health care services meeting the client’s/patient’s needs	Patient experience	Patient experience
Accessibility	Wait time for immediate care for minor health problem	Access to team-based primary care	Access to team-based primary care
Appropriateness	Child immunization Colon cancer screening Breast cancer screening Smoking cessation advice in primary health care Influenza immunization, 65+ Well-baby screening Blood pressure testing Screening for modifiable risk factors in adults with coronary artery disease Screening in adults with diabetes Screening for modifiable risk factors in adults with hypertension Treatment of dyslipidemia Treatment of acute myocardial infarction Treatment of anxiety	Disease tracking	
Comprehensiveness	Primary health care support for self-management of chronic conditions		
Coordination	Primary health care team effectiveness score	Intra-agency referrals Co-treatment Communication Team processes Patient care plan Intersectoral linkages/partnership	Referrals – within and outside of primary health care Co-treatment Care coordination
Effectiveness	Blood pressure for hypertension	Patient health status	Patient health status Patient goals
Efficiency	Unnecessary duplication of medical tests reported by provides		
Governance	Maintaining medication ad problem list in primary health care		
Health Status	Overweight and obesity rate		
IT infrastructure	Uptake of information and communication technology in primary health care organizations		
Workforce	Primary health care provider full-time equivalents	Staff experience Workload measurement	Staff experience Workload measurement

CIHI = the Canadian Institute of Health Information.

Although collaborative processes are important to consider from a performance indicator perspective, fundamentally team-based primary health care is a model of primary health care designed to expand the traditional biomedical approach by bringing in multiple perspectives to assist in addressing the broader determinants of health. However, the current indicators and mechanisms of assessing the quality of care in teams remain narrow in scope and focus, primarily on individual behaviors closely aligned with a biomedical model of care (e.g. medication review, diabetes complications) (Health Quality Ontario, 2015) and have yet to catch up with this shift to comprehensive, integrated primary health care services.

It must be recognized that team-based models are only a small subset of primary health care services (Glazier *et al.*, 2015), but determining appropriate indicators will support quality care and help ensure those individuals who would most benefit from this model of care receive the appropriate services. The World Health Organization’s International Classification of Functioning (WHO-ICF) (World Health Organization, 2013) might offer a framework to support future work in measuring and evaluating interprofessional primary health care. The WHO-ICF is an internationally recognized perspective that recognizes the role of personal, social and contextual factors in optimizing function and health (Dufour and Lucy, 2010). It is an ideal framework in which to situate interprofessional collaboration and would assist in broadening the approach to measurement in primary health care in Canada. The WHO-ICF could easily be applied to primary health care practices thereby providing a universal, philosophical foundation, and approach to interprofessional primary health care across various practice models and settings. The WHO-ICF (World Health Organization, 2013) also aligns well with popular approaches to health promotion and chronic disease management such as the expanded chronic care model (Barr *et al.*, 2003).

Quality improvement was identified as one of the top processes to support interprofessional primary health care teams, and the literature has shown that quality improvement processes such as audit and feedback (Ivers *et al.*, 2012) lead to enhanced quality health care. Within team-based primary health care, a study by Donnelly *et al.* (2016) used a participatory evaluation approach that involved primary health care team members not only identifying their own program outcomes but receiving regular feedback on their performance during team process meetings. Team members specifically highlighted the use of such an evaluation approach to build cohesion among the team.

Interestingly quality improvement was also identified as an assessment, outcome, and/or indicator to measure the value of care. The research team removed these responses from the analysis as quality improvement was considered to be the actual process in which teams are measured not the indicator itself. However, it could, in fact, be argued that the nature, depth or extent of engagement in quality improvement may in fact be a performance indicator for quality in team-based primary health care. Research from the field or program evaluation and their work examining ‘process use’ (Patton, 1998) could inform an exploration of how quality improvement itself could be formulated to be an indicator for primary health care teams. However, these results also point to the fact that determining what is an indicator for primary health care teams is very difficult. How teams operationalize these concepts are challenging, with providers coming from a diverse set of professional backgrounds and providing care to the broadest range of clients and conditions. In this study, participants responded with almost three times greater frequency when asked about characteristics of collaboration as compared to questions related to identifying outcomes, indicators, or assessments of team-based primary health care.

### Limitations

The authors acknowledge limitations to the study that include the use of a convenience sample of participants’ attending the pre-conference stakeholder meeting and aggregate data that does not allow for granular level of data analysis by profession. Different facilitators led the stakeholder meetings and therefore there may have been differences in how the questions were discussed. Next steps of this work are to develop mechanisms to identify data at the patient encounter level for providers of primary health care teams to further inform and develop indicators.

## Conclusion

With the growing emphasis on primary health care teams, it is critical that measures that capture the value of the team include indicators that teams feel they can directly act or contribute to. The study is a critical step in this process as it offers data from the interprofessional team members on both current and potential performance measurement. Team members specifically identified the need to include a team process indicator as well an indicator that focuses on global health status as opposed to the current biomedical indicators. While there are many frameworks to measure primary health care quality, these have relied on delivery models where primary health care is largely provided by solo practitioners. With an aging population and growing emphasis on chronic disease management, it will be imperative for comprehensive, interprofessional primary health care teams to turn to frameworks that consider health within a broader lens, where quality of life, function, and participation become important outcomes.

## Supplementary material

For supplementary material accompanying this paper visit https://doi.org/10.1017/S1463423619000409. click here to view supplementary material
