# Supplementary material for: Measuring the performance of interprofessional primary health care teams: understanding the teams perspective
Source: Prim Health Care Res Dev. 2019 Aug 28;20:e125. doi: 10.1017/S1463423619000409 (PMC6719251; doi:10.1017/S1463423619000409)
Supplement: Supplementary file 1 [file S1463423619000409sup.zip › S1463423619000409sup001.docx]

Interprofessional Health Provider Stakeholder Meeting

**Purpose: Measure and demonstrate value of IP collaborative primary care**

Within this session we are interesting in learning about:

1) How each of the interdisciplinary health professions working in Family Health Teams contribute to collaborative patient care.

2) The measures/indicators/outcomes **currently** being used to demonstrate the value of this contribution

3) The measures/indicators/outcomes that could be used to demonstrate the value of their contribution In the **future.**

Working in small groups of 10 or less for both Part A and B.

- If the room has multiple professions, we will ask professions to sit together.
- In small groups individuals will first document their responses to questions on the worksheet provided.
- A self-identified group leader will facilitate a discussion of the individually responses of the group and pull together the broad themes. Themes will be documented on poster paper provided to each team.
- The room facilitator will call upon each of the group leaders to present the themes identified in their groups and provide an overall summary of the groups.

Documentation:

- Each individual will submit their worksheets
- Each group leader will submit their themes – poster paper
- Each facilitator will submit their overall room summary

**PART A: What is Currently Happening**

1. What does interprofessional collaboration look like at your FHT? Please name some of the key elements of collaboration at your FHT.

|  |
| --- |

1. What measures/indicators/outcomes are currently being used to demonstrate the **value of interprofessional collaboration** at your FHT? In relation to your profession, please describe these as specifically as possible?

|  |
| --- |

1. What measures/indicators/outcomes are currently being used to demonstrate the **value of your profession** at your FHT that are **different than those used to measure the value of interprofessiona**l collaboration? In relation to your profession, please describe these as specifically as possible?

|  |
| --- |

**PART B: Future Possibilities**

**YOUR PROFESSION**

1. What other measures/indicators/outcomes (that are not currently in place) could help demonstrate your profession’s value in a collaborative team environment to the a) individual patient, b) within the team/FHT and, c) broader population?

| **At the individual patient encounter** | **Within the team and FHT** | **Within the broader population.** |
| --- | --- | --- |
|  |  |  |

**INTERPROFESSIONAL TEAM COLLABORATION**

1. What other measures/indicators/outcomes (that are not currently in place) could help demonstrate the value of collaboration to the a) individual patient, b) within the team/FHT and, c) broader population?

| **At the individual patient encounter** | **Within the team and FHT** | **Within the broader population.** |
| --- | --- | --- |
|  |  |  |
