# Supplementary material for: Measuring the performance of interprofessional primary health care teams: understanding the teams perspective
Source: Prim Health Care Res Dev. 2019 Aug 28;20:e125. doi: 10.1017/S1463423619000409 (PMC6719251; doi:10.1017/S1463423619000409)
Supplement: Supplementary file 1 [file S1463423619000409sup.zip › S1463423619000409sup002.docx]

Interprofessional Health Provider Stakeholder Meeting

Thank you for being here today and your willingness to engage in a discussion on interprofessional collaboration. We hope this is an informative and engaging session for you.

In our small groups we will discuss two broad questions on the topic of measuring and demonstrating value of interprofessional collaboration: a) What is currently happening; and, b) What are the future possibilities?

The purpose of this activity is to provide you with an opportunity to engage in the topic, as well as to conduct research so that findings that come out of this discussion can be of use to you and the AFHTO membership in advancinginterprofessional collaboration.

Worksheets with the activity questions will be provided to all participants – please record your own thoughts on this sheet. These worksheets collected at the end of the session in order to explore themes that arise from the discussions. Findings from today’s session may be used in a future presentation, article, and report. Your confidentiality in this will be maintained. We are asking that participants hand in their worksheets at the end of the session, however, you are free not to if you prefer. Worksheets will be kept for ten years and then will be destroyed.

Group discussions will be recorded on flip chart paper. Flip chart paper will also be collected at the end of the session, and will be used in the same manner as the worksheets. They will also be kept for ten years and then destroyed.

Once all the data are collected and analyzed for this project, AFHTO plans on sharing this information with the AFHTO membership and research community through reports, seminars, conferences, presentations, and journal articles.

If you have any questions about the study, please do not hesitate to contact improve@afhto.ca. This project is part of a larger study that received ethics clearance through ____ Research Ethics Committee. Should you have any comments or concerns resulting from your participation in this study, please contact Insert name of ethics officer.

Thank you for your involvement.
